# Tempol Supplementation Restores Diaphragm Force and Metabolic Enzyme Activities in *mdx* Mice

**DOI:** 10.3390/antiox6040101

**Published:** 2017-12-06

**Authors:** David P. Burns, Izza Ali, Clement Rieux, James Healy, Greg Jasionek, Ken D. O’Halloran

**Affiliations:** Department of Physiology, University College Cork, Western Gateway Building, Western Road, Cork T12 XF62, Ireland; david.burns@umail.ucc.ie (D.P.B.); izza_sunflower@hotmail.com (I.A.); c.rieux@stanbiotec.com (C.R.); 113380546@umail.ucc.ie (J.H.); g.jasionek@ucc.ie (G.J.)

**Keywords:** Duchenne muscular dystrophy, *mdx*, tempol, oxidative stress, diaphragm, antioxidant

## Abstract

Duchenne muscular dystrophy (DMD) is characterized by striated muscle weakness, cardiomyopathy, and respiratory failure. Since oxidative stress is recognized as a secondary pathology in DMD, the efficacy of antioxidant intervention, using the superoxide scavenger tempol, was examined on functional and biochemical status of dystrophin-deficient diaphragm muscle. Diaphragm muscle function was assessed, ex vivo, in adult male wild-type and dystrophin-deficient *mdx* mice, with and without a 14-day antioxidant intervention. The enzymatic activities of muscle citrate synthase, phosphofructokinase, and lactate dehydrogenase were assessed using spectrophotometric assays. Dystrophic diaphragm displayed mechanical dysfunction and altered biochemical status. Chronic tempol supplementation in the drinking water increased diaphragm functional capacity and citrate synthase and lactate dehydrogenase enzymatic activities, restoring all values to wild-type levels. Chronic supplementation with tempol recovers force-generating capacity and metabolic enzyme activity in *mdx* diaphragm. These findings may have relevance in the search for therapeutic strategies in neuromuscular disease.

## 1. Introduction

Duchenne muscular dystrophy (DMD) is the most common form of inherited muscle disease in childhood, with an estimated incidence of 1:3500 male births [1]. DMD is caused by a deficiency in the protein dystrophin, which is a component of the dystrophin associated protein complex (DAPC) [2]. The DAPC has a structural role in linking the actin cytoskeleton to the extracellular matrix, thus stabilizing the sarcolemma during muscle contraction and relaxation [3]. Dystrophin deficiency results in destabilization of the DAPC, leading to muscle weakness and fragility, resulting in muscle damage, fibrosis, and necrosis [4]. Inflammation is secondary to muscle damage in DMD, with attendant disruption to Ca^2+^ homeostasis, oxidative stress, and mitochondrial dysfunction [5]. DMD patients suffer severe limb and respiratory muscle weakness [6]. Patients have compromised lung function due to diaphragm muscle weakness, altered chest wall compliance, and scoliosis [7]. Trans-diaphragmatic pressures are low in DMD boys [8], and disordered breathing can occur, particularly during sleep, leading to obstructive sleep apnea and hypoventilation [9]. Cardio-respiratory failure is the leading cause of death in DMD.

Oxidative stress is the result of increased reactive oxygen species (ROS) and/or decreased antioxidant capacity. ROS, in particular superoxide anions, are highly chemically reactive substances that can react with nucleic acids, lipids, and proteins, thus hindering cellular metabolism, resulting in cell injury [10]. Low levels of ROS are essential for optimal force production during muscle contraction, and act as important physiological signaling molecules that alter enzymatic activity and gene expression [11]. Large amounts of ROS have deleterious effects on muscle force and endurance, such as during strenuous exercise and chronic disease states, such as muscular dystrophy [12,13,14].

The most widely studied preclinical model of DMD is the dystrophin-deficient *mdx* mouse. Diaphragm muscle from *mdx* mice displays similar characteristics to DMD patients, including loss of function [15], muscle fibrosis and necrosis [16], inflammation, and oxidative stress [17]. Respiratory deficits are present in the *mdx* mouse, including hypoventilation [18]. An imbalance between ROS production and antioxidant scavenging capacity in muscle can promote muscle damage and mitochondrial dysfunction, with resultant impaired muscle function. Antioxidant capacity and estimates of ROS turnover have been examined in a number of tissues from *mdx* mice, including skeletal and cardiac muscle, and the brain. Markers of oxidative stress are increased in hearts from *mdx* mice [19], as well as in limb and diaphragm muscle [20,21,22]. Increased superoxide dismutase activity is reported in *mdx* cerebellum and prefrontal cortex, and this increase in antioxidant activity was associated with decreased lipid peroxidation, suggesting a protective role of superoxide dismutase in *mdx* mouse brain [23].

Disruption of the DAPC in DMD results in nitric oxide synthase (NOS) displacement from the sarcolemma [3]. NOS has a physiological role in the synthesis of nitric oxide (NO), which exerts protective effects in muscle in response to cell injury. Activity of the superoxide generating enzyme complex nicotinamide adenine dinucleotide phosphate-oxidase (NOX) is increased in *mdx* muscle [21,24]. Pharmacological inhibition of NOX, using apocynin, improved calcium handling and contractility in *mdx* hearts [24]. Similarly, inhibition of ROS with diapocynin reduced ROS production and prevented force loss induced by eccentric contractions in dystrophic limb muscle [25]. Increased ROS can activate pro-inflammatory pathways, resulting in inflammation and fibrosis, and can promote Ca^2+^ dysregulation. Recent data suggest a complex interaction between oxidative stress, Ca^2+^ dysregulation, and inflammation as secondary mediators of pathology in DMD [5,26,27].

Currently no cure exists for DMD, therefore, much work is focused on discovering novel therapies for the treatment of the disease. In the present study, we used the *mdx* mouse model of DMD to investigate the therapeutic potential of tempol on diaphragm muscle force and oxidative and glycolytic metabolic enzyme activities. Tempol is a membrane permeable superoxide dismutase mimetic, which exerts antioxidant effects by scavenging superoxide anions. Tempol acts by catalyzing the reaction of superoxide into oxygen or hydrogen peroxide. Previous work has shown that tempol exerts a positive inotropic effect on rat pharyngeal dilator muscle function ex vivo [28], and prevents hypoxia-induced muscle weakness [29,30] and oxidative stress [31,32]. It is also established that tempol prevents temperature-dependent force loss in mouse and rat muscle ex vivo [33]. We hypothesized that chronic antioxidant therapy in *mdx* mice would improve diaphragm muscle function.

## 2. Materials and Methods

### 2.1. Ethical Approval

Procedures involving live animals were performed under license in accordance with Irish and European legislation, following prior approval by University College Cork’s animal research ethics committee. The approval number from University College Cork’s animal research ethics committee is AEEC 2013/035.

### 2.2. Experimental Animals

Male and female wild-type (C57BL/10ScSnJ) and *mdx* (C57BL/10ScSn-Dmd^mdx^/J) mice were purchased from the Jackson Laboratory (Jackson Laboratory, Bar Harbor, ME, USA) and bred at University College Cork’s animal housing facility. Animals were housed conventionally in a temperature- and humidity-controlled facility, operating on a 12 h light–12 h dark cycle with food and water available ad libitum. Male mice were studied at 14 weeks of age, and were assigned to four groups: wild-type (26.2 ± 1.6 g; *n* = 7), *mdx* (29.2 ± 2.8 g; *n* = 7), *mdx* + tempol in vitro (32.2 ± 2.5 g; *n* = 7) and *mdx* + tempol in vivo (30.1 ± 2.2g; *n* = 9); *mdx* animals were randomly assigned to groups. The *mdx* + tempol in vivo group received tempol (1 mM 4-hydroxy-TEMPO; Sigma Aldrich, Wicklow, Ireland) in their drinking water for two weeks (from 12 to 14 weeks of age), equivalent to a dose of 20–35 mg/g body weight, taking an estimate of fluid intake and known body mass gain over the intervention period. The dose was informed by previously published work in rat, wherein tempol proved efficacious in preventing respiratory muscle dysfunction in response to redox stress associated with exposure to chronic intermittent hypoxia [29,30].

### 2.3. Muscle Physiology

#### 2.3.1. Ex Vivo Muscle Preparation

Animals were anaesthetized with 5% isoflurane in air, and euthanized by cervical dislocation. Diaphragm muscle was immediately excised, with central tendon and rib intact for functional studies. Additional diaphragm muscle samples were snap frozen in liquid nitrogen and stored at −80 °C for later analysis. Diaphragm muscle was suspended vertically in a water-jacketed tissue bath at 35 °C, filled with Krebs solution (in mM: 120 NaCl, 5 KCl, 2.5 Ca^2+^ gluconate, 1.2 MgSO_4_, 1.2 NaH_2_PO_4_, 25 NaHCO_3_ and 11.5 glucose) and d-tubocurarine (25 μM). Preparations were equilibrated with hyperoxic (95% O_2_/5% CO_2_) gas. The rib end of the preparation was attached to an immobile hook at the base of a muscle holder, and the central tendon was attached to a dual mode lever transducer system (Aurora Scientific Inc., Aurora, ON, Canada) with non-elastic string, to allow the assessment of isometric and isotonic contractile properties. For the *mdx* + tempol in vitro group, diaphragm muscle from *mdx* mice was studied in Krebs solution containing 1 mM tempol, which has been shown to exert positive inotropic effects on rat respiratory muscle in isolated muscle preparations [28,29]).

#### 2.3.2. Isometric Protocol

The muscle strips were stimulated via field stimulation with platinum electrodes at supramaximal voltage. Optimal length (L_o_), the length producing maximum twitch force, was obtained by repeated isometric twitch stimulation (supramaximal voltage; 1 ms duration) at varying muscle lengths, achieved by adjustment of a micro-positioner. The L_o_ was recorded, and the muscle remained at this length for the remainder of the experiment. Following a 10 min equilibration period, contractile properties were examined. First, a single twitch was elicited, and twitch force, contraction time (CT; time to peak force), and half-relaxation time (½ RT; time for force to decay by 50%) were determined. Next, an isometric tetanic contraction was elicited by stimulating muscle strips with supramaximal voltage at 100 Hz for 300 ms duration. Peak isometric tetanic force (F_max_) was determined [34,35].

#### 2.3.3. Isotonic Protocol

Concentric contractions were evoked at varying loads in an incremental step-test (0%, 5%, 10%, 20%, 30%, 40%, 50%, 60%, 80%, 100%; % of F_max_). Each contraction was interspaced by 1 min, and muscle length returned to L_o_ following each contraction. Total shortening was considered the maximum distance shortened during contraction; shortening velocity was measured during the initial 30 ms of shortening [34,36]. Mechanical work (force *x* total shortening) and power (force *x* shortening velocity) were measured at each % load [34,37].

### 2.4. Muscle Biochemistry

#### 2.4.1. Tissue Preparation

Diaphragm samples stored at −80 °C were removed and allowed to defrost at 4 °C for 5 min. All procedures were performed at 4 °C to prevent protein degradation. Samples were homogenized in a lysis buffer (RIPA) made up from 10× RIPA, deionized water, 200 mM sodium fluoride, 100 mM phenylmethylsulfonyl fluoride, protease cocktail inhibitor 1, and phosphatase cocktail inhibitor 2. Following the homogenization process, the reactant mixtures were centrifuged (15,339× *g*) and the supernatants were harvested. Total amount of protein for each tissue sample was determined using Pierce^®^ Bicinchoninic Acid Assay (BCA assay, Thermo Scientific, Fisher, Dublin, Ireland). Supernatants were aliquoted and stored at −80 °C for future use.

#### 2.4.2. Metabolic Enzyme Assays

Tissue homogenates were used for enzymatic activity assays. The experimental procedures for citrate synthase, phosphofructokinase, and lactate dehydrogenase activity assays were performed in accordance with the technical bulletins of a citrate synthase assay kit (CS0720; Sigma-Aldrich, Wicklow, Ireland), phosphofructokinase activity colorimetric assay kit (MAK093; Sigma-Aldrich), and lactate dehydrogenase activity assay kit (MAK066; Sigma-Aldrich), respectively. Results are presented as enzymatic activity per 1 mg of protein in tissue homogenate.

### 2.5. Data Analysis

Specific force was normalized for muscle tissue cross-sectional area (CSA), and calculated in N/cm^2^. Muscle CSA was estimated for each muscle strip by dividing the muscle mass (weight in grams) by the product of muscle L_o_ (cm) and muscle density (assumed to be 1.06 g/cm^3^). For isotonic load relationships, data were expressed as the measured parameter versus % load. Total muscle shortening was normalized to L_o_ and expressed in L_o_/s. Maximal total shortening (S_max_) and maximum shortening velocity (V_max_) occurred at 0% load. Maximum mechanical work (W_max_) and power (P_max_) occurred at ~30–40% load.

### 2.6. Statistical Analysis

Values are expressed as mean ± SD or are represented graphically as box and whisker plots (median, 25th–75th centile, and minimum and maximum). Data were statistically compared by unpaired Student’s *t* tests with Welch’s correction where appropriate, and two-way analysis of variance (ANOVA); for data from incremental load tests). *p* < 0.05 was deemed to be statistically significant.

## 3. Results

### 3.1. Isometric Force and Twitch Contractile Kinetics

**Figure 1:**
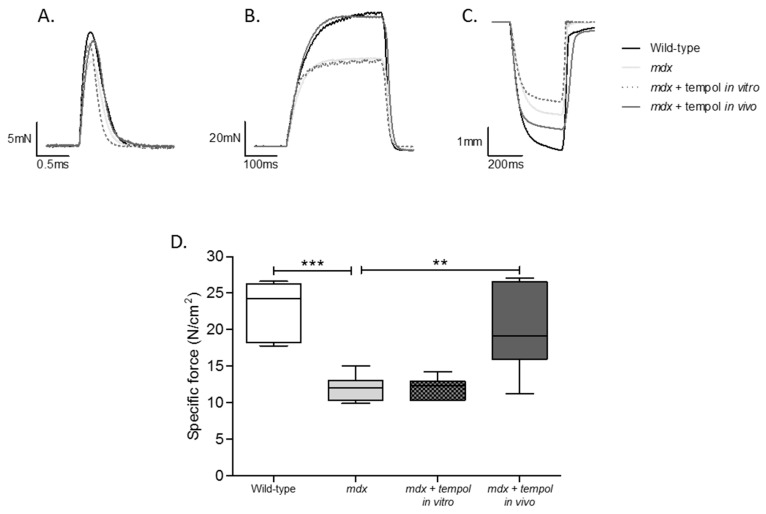
Peak Isometric Tetanic Force. Representative traces for muscle twitch (**A**) and tetanic (**B**) contractions and maximum unloaded shortening (**C**) for diaphragm muscle from wild-type, *mdx*, *mdx* + tempol in vitro and *mdx* + tempol in vivo. Group data for diaphragm muscle peak tetanic force (**D**) from wild-type (*n* = 7), *mdx* (*n* = 7), *mdx* + tempol in vitro (*n* = 7) and *mdx* + tempol in vivo (*n* = 8). For the *mdx* + tempol in vitro group, diaphragm muscle preparations were studied in Krebs solution containing 1 mM tempol in vitro. The *mdx* + tempol in vivo group received 1 mM tempol in their drinking water for two weeks. Values are expressed as box and whisker plots (median, 25–75% centiles and minimum and maximum values) and data were statistically compared by Student’s *t* tests. *** *p* < 0.0001; ** *p* = 0.0069.

Representative original traces for diaphragm muscle twitch (A) and tetanic (B) contractions and maximum unloaded shortening (C) are shown in Figure 1 for wild-type, *mdx*, *mdx* + tempol in vitro and *mdx* + tempol in vivo. Table 1 shows data for diaphragm muscle twitch force and contractile kinetics from all four groups. Twitch force was significantly lower in *mdx* compared with wild-type diaphragm (*p* = 0.0066, Student’s *t* test). There was a significant decrease in diaphragm ½ RT following tempol administration in vivo in *mdx* mice (*p* = 0.0046). Twitch force was significantly higher in *mdx* + tempol in vivo compared with *mdx* (*p* = 0.0111). F_max_ was significantly lower in *mdx* diaphragm compared with wild-type (*p* < 0.0001; Figure 1D). F_max_ was significantly higher in *mdx* + tempol, in vivo, compared with *mdx* (*p* = 0.0069), such that values were equivalent to wild-type values for peak force generation. Bath application of tempol to *mdx* diaphragm had no effect on force-generating capacity or contractile kinetics compared with *mdx* (Figure 1 and Table 1).

### 3.2. Isotonic Contractile Parameters and Kinetics

Table 1 shows data for diaphragm muscle isotonic contractile parameters. W_max_ was significantly lower in *mdx* compared with wild type diaphragm (*p* = 0.0276; Student *t* test). W_max_ was significantly elevated in *mdx* + tempol in vivo compared with *mdx* (*p* = 0.0085). P_max_ was lower in *mdx* diaphragm compared with wild-type, but this did not reach statistical significance (*p* = 0.0709). P_max_ was significantly elevated in *mdx* + tempol in vivo compared with *mdx* (*p* = 0.0217), completely restoring P_max_ to wild-type values. No significant differences were noted for S_max_ between groups. Bath application of tempol to *mdx* diaphragm significantly decreased V_max_ compared with *mdx* (*p* = 0.0353), but had no effect on W_max_ or P_max_.

### 3.3. Isotonic Load Relationships

**Figure 2:**
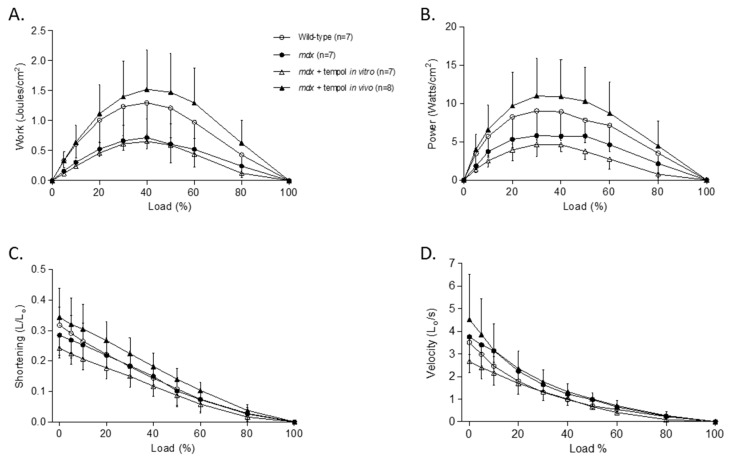
Diaphragm Muscle Isotonic Contractile Properties. Group data (mean ± SD) for work—(**A**), power—(**B**), shortening—(**C**) and shortening velocity—(**D**) load relationships for diaphragm muscle from wild-type (*n* = 7), *mdx* (*n* = 7), *mdx* + tempol in vitro (*n* = 7) and *mdx* + tempol in vivo (*n* = 8). For the *mdx* + tempol in vitro group, diaphragm muscle preparations were studied in Krebs solution containing 1 mM tempol in vitro. The *mdx* + tempol in vivo group received 1 mM tempol in their drinking water for two weeks. Data were statistically compared by two-way analysis of variance (ANOVA). Work: Load: *p* < 0.0001; Gene: *p* = 0.0071; tempol in vitro: *p* = 0.5020; tempol in vivo: *p* = 0.0063; Power: Load: *p* < 0.0001; Gene: *p* = 0.0115; tempol in vitro: *p* = 0.0037; tempol in vivo: *p* = 0.0177; Shortening: Load: *p* < 0.0001; Gene: *p* = 0.7068; tempol in vitro: *p* = 0.0995; tempol in vivo: *p* = 0.1117; Velocity: Load: *p* < 0.0001; Gene: *p* = 0.1756; tempol in vitro: *p* = 0.0159; tempol in vivo: *p* = 0.5427.

Figure 2A–D shows data for diaphragm muscle isotonic load relationships. Loading had a significant effect on work (*p* < 0.0001; two-way ANOVA; Figure 2A), power (*p* < 0.0001; Figure 2B), shortening (*p* < 0.0001; Figure 2C) and shortening velocity (*p* < 0.0001; Figure 2D) for diaphragm muscle from all four groups. Work (*p* = 0.0071) and power production (*p* = 0.0115) were significantly reduced in *mdx* diaphragm compared with wild-type. Tempol supplementation in vivo in *mdx* mice significantly increased work (*p* = 0.0063) and power (*p* = 0.0177) production compared with *mdx*. Bath application of tempol significantly reduced *mdx* diaphragm power production (*p* = 0.0037) and shortening velocity (*p* = 0.0159) compared with *mdx*.

### 3.4. Metabolic Enzyme Activity

**Figure 3:**
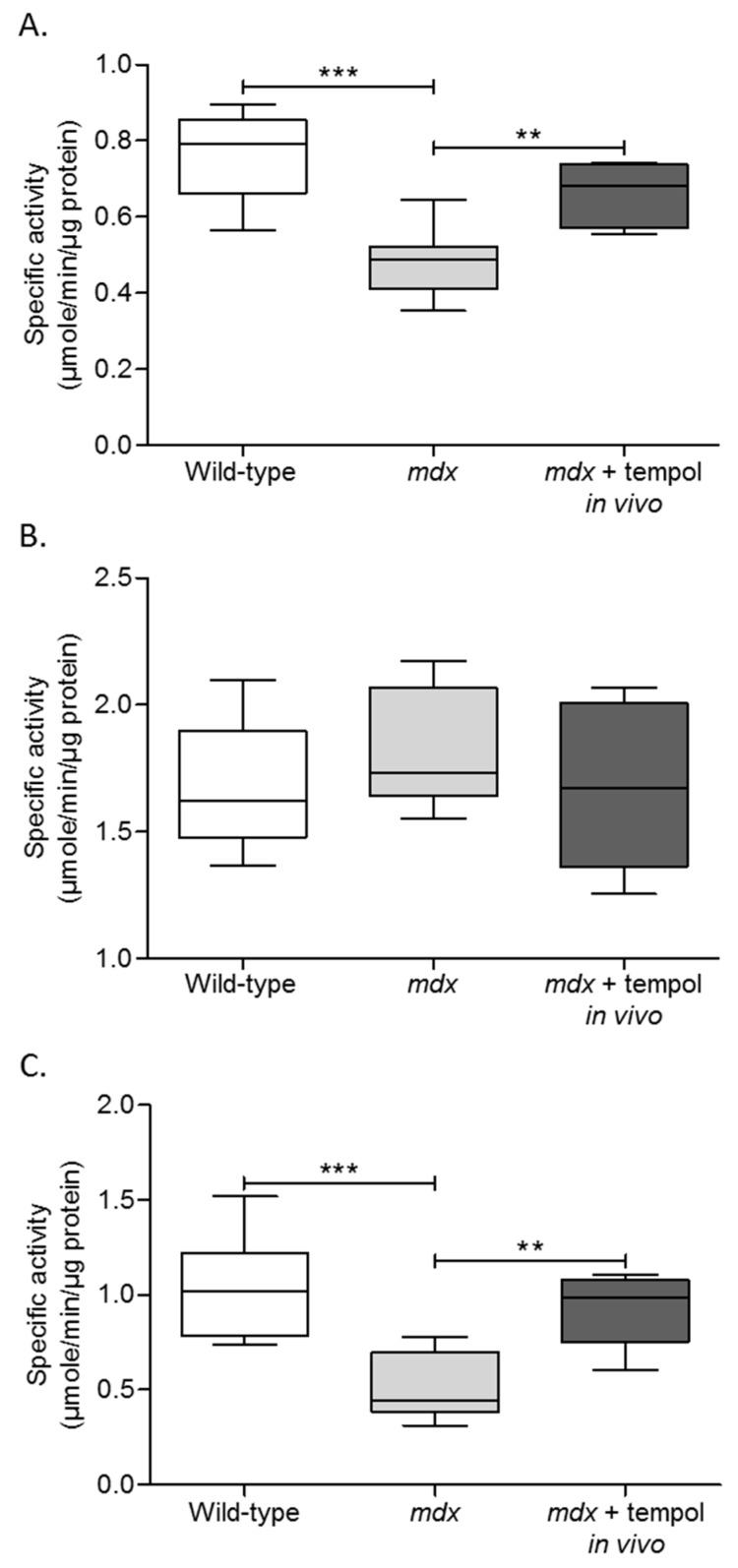
Metabolic Enzyme Activities. Group data for citrate synthase (**A**), phosphofructokinase (**B**), and lactate dehydrogenase (**C**) enzyme activities in diaphragm muscle from wild-type, *mdx* and *mdx* + tempol in vivo. *mdx* + tempol in vivo received 1 mM tempol in their drinking water for two weeks. Values are expressed as box and whisker plots (median, 25–75% centiles and minimum and maximum values), and data were statistically compared by unpaired Student’s *t* tests. (**A**) *** *p* = 0.0003; ** *p* = 0.005. (**C**) *** *p* = 0.001; ** *p* = 0.0018.

Figure 3A–C shows data for diaphragm muscle metabolic enzyme activities. Citrate synthase activity was significantly lower in *mdx* diaphragm compared with wild-type (*p* = 0.0003; unpaired Student’s *t* test; Figure 3A). Chronic tempol supplementation in *mdx* significantly increased diaphragm citrate synthase activity compared with *mdx* (*p* = 0.005). No significant differences were noted between groups for diaphragm phosphofructokinase activity (Figure 3B).

Lactate dehydrogenase activity was significantly lower in *mdx* diaphragm compared with wild-type (*p* = 0.001; Figure 3C). Chronic tempol supplementation in *mdx* significantly increased diaphragm lactate dehydrogenase activity compared with *mdx* (*p* = 0.0018).

## 4. Discussion

The main findings of this study are (1) diaphragm muscle weakness in *mdx* mice is evidenced by reduced specific force, work and power output; (2) dystrophin-deficiency in *mdx* diaphragm is associated with reduced citrate synthase and lactate dehydrogenase enzyme activities, whereas phosphofructokinase activity is equivalent to wild-type; (3) chronic tempol supplementation in *mdx* mice completely restored diaphragm force- and power-generating capacity; (4) chronic tempol supplementation significantly increased *mdx* diaphragm citrate synthase and lactate dehydrogenase enzyme activities to wild-type levels; (5) acute bath application of tempol had limited effects on dystrophic diaphragm function ex vivo.

Diaphragm muscle thickening and fat accumulation are described in DMD patients, associated with diaphragm muscle weakness [38,39]. Ventilatory capacity decreases with age, since DMD is a progressive disease [8,40]. Respiratory failure is a leading cause of mortality in DMD. Although DMD is primarily caused by a genetic abnormality resulting in the absence of the dystrophin protein, many secondary pathologies have been identified as contributing to the muscle pathology observed in DMD, including inflammation, Ca^2+^ dysregulation, and redox stress [5,26].

Diaphragm muscle degeneration, fibrosis, and dysfunction are reported in *mdx* mice, which display an overt pathology similar to human DMD [16]. In the present study, we report diaphragm weakness in *mdx* mice consistent with other reports [15,16,41]. Diaphragm weakness in *mdx* was characterized by reduced twitch and tetanic muscle force and reduced W_max_. Diaphragm muscle mechanical work and power generation were reduced across the load continuum, revealing severe intrinsic mechanical dysfunction in dystrophic diaphragm muscle. These functional deficits were associated with altered metabolic enzyme activities, characterized by reduced citrate synthase and lactate dehydrogenase activity. Citrate synthase catalyzes the first step within the Krebs cycle, the condensation of acetyl-coenzyme A with oxaloacetate to form citrate, and serves as a marker of the mitochondrial matrix. Reduced citrate synthase activity suggests reduced mitochondrial activity, and hence, aerobic capacity in *mdx* diaphragm. This reduction in mitochondrial activity may relate to a loss of mitochondrial content, resulting in reduced oxidative capacity, or may be due to oxidative stress-induced mitochondrial dysfunction or mitophagy. Phosphofructokinase catalyzes the phosphorylation of fructose-6-phosphate to fructose-1,6-bisphosphate, thus functioning as a key regulatory step in the glycolytic pathway. Phosphofructokinase activity was unchanged in *mdx* diaphragm compared with wild-type. Lactate dehydrogenase catalyzes the forward and backward conversion of pyruvate to lactate, with the skeletal muscle isoform kinetically favoring conversion from pyruvate to lactate. Since glycolysis, which diverts pyruvate to lactate, serves to diminish redox stress [42], reductions in lactate dehydrogenase activity can cause oxidative stress associated with increased cellular oxygen consumption [43]. Metabolic reprogramming in *mdx* and DMD muscle is complex, and likely temporally regulated in dynamic fashion. Indeed, contrary to our observation, a recent report revealed elevated activity in oxidative enzymes of the Krebs cycle in *mdx* diaphragm [44].

Indicators of oxidative stress, such as 4-hydroxynonenal (4-HNE) and dihydroethdium (DHE), are increased in *mdx* diaphragm, revealing increased oxidative stress which likely has functional implications [17]. Antioxidant enzymes, such as superoxide dismutase, catalase and glutathione peroxidase, are elevated in limb muscle from *mdx* mice [20], which may contribute to the mild limb muscle phenotype which is observed in *mdx* mice compared with the diaphragm muscle, which more closely represents the human diaphragm pathology. Studies have shown that NOX subunits and superoxide production are increased in skeletal muscle of *mdx* mice [21,45]. Oxidative stress contributes to cardiomyocyte dysfunction in dystrophic hearts, and NOX2 inhibition resulted in restoration of calcium handling and contractility, and reduced collagen expression in cardiomyocytes [19,24]. The NOX subunits gp91^phox^, p67^phox^ and Rac1 are increased in the tibialis anterior of *mdx* mice, associated with increased superoxide production [21].

Tempol has previously been shown to have positive inotropic effects on rat pharyngeal dilator (respiratory) muscle performance ex vivo [28,29]. Interestingly, tempol was shown to ameliorate upper airway muscle weakness in a rat model of chronic intermittent hypoxia [29]. In a similar model of chronic intermittent hypoxia, tempol exerted modest effects on diaphragm muscle force, but reversed chronic intermittent hypoxia-induced diaphragm fatigue [30]. Chronic tempol supplementation has also been shown to prevent sustained hypoxia induced pharyngeal dilator muscle weakness [31], but not diaphragm muscle dysfunction [37]. Collectively, these studies illustrate that tempol can exert inotropic effects on rodent respiratory muscle and ameliorate muscle weakness in models of hypoxic stress via superoxide scavenging, although this capacity appears muscle specific and may be dependent on the mode of hypoxia (sustained versus intermittent). This may have relevance to putative hypoxia-dependent remodeling in *mdx* muscle. DMD patients [9,46] and *mdx* mice [47,48] hypoventilate. DMD patients exhibit nocturnal hypoxemia and sleep-disordered breathing [49,50], and exposure to chronic intermittent hypoxia has been shown to further weaken *mdx* diaphragm [51]. Therefore, hypoxic stress may be implicated in *mdx* and DMD pathology, such that antioxidant strategies (such as tempol), proven to prevent hypoxia-dependent respiratory muscle weakness and fatigue, may be especially useful.

Since elevated superoxide production is reported in the diaphragm muscle of *mdx* mice, we sought to examine the efficacy of the superoxide scavenger tempol on *mdx* diaphragm muscle function. To our knowledge, this is the first report on the effects of tempol on muscle function and metabolic enzyme activity in the *mdx* mouse. Chronic tempol supplementation resulted in increased diaphragm force generation, revealed both in twitch and tetanic contractions, impressively restoring these functional indices to values equivalent to wild-type values. Work- and power-generating capacity expressed as a function of load were significantly increased in diaphragm from tempol supplemented mice compared with *mdx*, resulting in a complete restoration of work- and power-generating capacity. W_max_ and P_max_ were similarly significantly increased. Interestingly, tempol decreased ½ RT for *mdx* diaphragm, suggesting an increased rate of calcium reuptake into the sarcoplasmic reticulum during muscle relaxation. These functional improvements in *mdx* diaphragm are likely attributable to scavenging of excessive ROS in *mdx* diaphragm [17,21,45], and improvements in calcium handling.

In the current study, the finding that acute bath application of tempol significantly reduced *mdx* diaphragm V_max_, demonstrates the functional significance of ROS signaling, most likely superoxide, within *mdx* diaphragm muscle preparations. Basal ROS are known to have physiological roles in cross-bridge cycling and muscle force generation [11]. However, elevated levels of ROS orchestrate a pro-oxidant environment, which can serve to impair cross-bridge cycling, and thus, reduce force production. A tonic inhibitory effect of ROS on respiratory muscle function, suppressing force-generating capacity, was revealed by the observation of positive inotropic effects of acute bath application of the superoxide scavengers, tempol, and tiron [28]. Moreover, H_2_O_2_ has been shown to cause concentration-dependent inotropic effects on rat respiratory muscle function, with high concentrations of ROS resulting in muscle weakness and fatigue, an effect blocked by catalase [52]. Notwithstanding the capacity for antioxidant scavenging to acutely affect muscle performance, the general lack of effect of bath application of tempol in *mdx* diaphragm is not surprising. We posit that chronically elevated levels of ROS in *mdx* diaphragm induce structural abnormalities that cannot be reversed by acute tempol application. Rather, it is evident that chronic antioxidant supplementation is required to re-establish redox homeostasis, returning ROS to physiological levels, limiting redox stress, allowing restoration of muscle function. The functional improvements in *mdx* diaphragm extended to restoration of citrate synthase and lactate dehydrogenase enzyme activities. Improved metabolic enzyme activity has obvious consequences for improved *mdx* diaphragm performance. We used enzyme activity as a surrogate marker for oxidative stress, since these proteins are often targets of redox stress, and can in turn be drivers of redox stress owing to alterations in bioenergetics. We interpret the restoration of aerobic and glycolytic enzyme activities as indirect evidence of the efficacy of tempol in ameliorating oxidative stress in *mdx* diaphragm, with the important added benefit of restoring energy homeostasis and ATP-generating capacity.

Previously, EUK-134, a novel catalytic mimetic of superoxide dismutase and catalase, was used to examine the role of oxidative stress in *mdx* diaphragm pathology. EUK-134 reduced markers of oxidative stress, inflammation, and indicators of muscle damage in *mdx* diaphragm, although only a partial rescue of diaphragm force was observed [53]. Treatment of *mdx* mice with ascorbic acid, an antioxidant and free radical scavenger, was shown to reduce creatine kinase levels, myonecrosis, inflammation, and the levels of 4-HNE [54]. These studies further highlight the close relationship between ROS and inflammation in dystrophic diaphragm. Beyond antioxidant effects, targeting ROS using antioxidant interventions in *mdx* mice may also have non-specific anti-inflammatory actions, further supporting their use as a potential therapeutic strategy in DMD. In respect of interventional studies in DMD, our data suggest that superoxide scavengers warrant attention. Tempol is not approved for use in humans, but dietary and/or pharmacotherapies mimicking its actions could prove beneficial in DMD. Many studies have revealed beneficial effects of antioxidants on muscle integrity (Table 2), but few studies have assessed the effects of antioxidants on diaphragm force generating capacity. It will be interesting to determine how tempol compares to previous studies in respect of improvements in diaphragm structure and quality.

The current study has relevance to therapeutic interventions in DMD, particularly those aimed at alleviating respiratory deficits in DMD boys. Since diaphragmatic weakness is observed in DMD boys, and likely contributes to breathing disturbances and the development of respiratory failure, strategies aimed at improving diaphragm muscle functional capacity in DMD are attractive. In respect of the current data set, it would be interesting in future studies to examine the effects of tempol supplementation on respiratory and cardiac function in *mdx* mice.

### Limitations

We acknowledge the current study employed a single dose of tempol at one time point in a progressive disease. However, notably, our intervention resulted in full recovery of diaphragm force. Future research should examine dose-dependent effects of tempol in *mdx* mice on respiratory and non-respiratory muscle structure and function. Mitochondrial function and direct measures of oxidative stress should be examined to determine the mechanism of force recovery in *mdx* diaphragm following tempol supplementation.

## 5. Conclusions

In conclusion, our findings show that tempol supplementation in *mdx* mice is efficacious, serving to restore diaphragm force- and power-generating capacity to levels equivalent to wild-type values. Functional improvements were accompanied by restoration of citrate synthase and lactate dehydrogenase enzyme activities in *mdx* diaphragm. Our study implicates superoxide anions and downstream ROS as pivotal mediators of dystrophin-deficient respiratory muscle pathophysiology. Recovery of diaphragm muscle contractile function was impressive in our study highlighting the potential utility of tempol and other superoxide scavengers in the treatment of DMD.

## Figures and Tables

**Table 1:** Diaphragm Muscle Contractile Properties.

	Wild-Type (*n* = 7)	*Mdx* (*n* = 7)	*mdx* + Tempol In Vitro (*n* = 7)	*mdx* + Tempol In Vivo (*n* = 8)	Student’s *t* Test
CT (ms)	18.0 ± 1.8	20.5 ± 4.5	17.9 ± 1.5	20.2 ± 3.1	$: *p* = 0.2196; †: *p* = 0.1796; £: *p* = 0.8777
½ RT (ms)	23.5 ± 0.6	23.5 ± 0.5	23.2 ± 0.2	17.8 ± 3.9	$: *p* = 0.9803; †: *p* = 0.1197; £: *p* = 0.0046
P_t_ (N/cm^2^)	5.1 ± 1.7	2.5 ± 0.7	2.7 ± 0.7	4.0 ± 1.2	$: *p* = 0.0066; †: *p* = 0.4821; £: *p* = 0.0111
W_max_ (J/cm^2^)	1.3 ± 0.5	0.7 ± 0.2	0.7 ± 0.4	1.5 ± 0.7	$: *p* = 0.0276; †: *p* = 0.6852; £: *p* = 0.0085
P_max_ (W/cm^2^)	9.0 ± 3.8	5.8 ± 0.9	4.7 ± 1.6	11.0 ± 4.9	$: *p* = 0.0709; †: *p* = 0.1329; £: *p* = 0.0217
S_max_ (L/L_o_)	0.32 ± 0.06	0.28 ± 0.07	0.24 ± 0.03	0.34 ± 0.10	$: *p* = 0.3457; †: *p* = 0.1532; £: *p* = 0.1936
V_max_ (L_o_/s)	3.5 ± 1.3	3.8 ± 0.8	2.7 ± 0.9	4.5 ± 2.0	$: *p* = 0.6766; †: *p* = 0.0353; £: *p* = 0.3433

Values (mean ± SD) for twitch contraction time (CT), twitch half-relaxation time (½ RT), peak twitch force (P_t_), maximum mechanical work (W_max_), maximum mechanical power (P_max_), peak shortening (S_max_), and peak shortening velocity (V_max_) of diaphragm muscle from the following groups: wild-type (*n* = 7), *mdx* (*n* = 7), *mdx* + tempol in vitro (*n* = 7) and *mdx* + tempol in vivo (*n* = 8). For the *mdx* + tempol in vitro group, diaphragm muscle preparations were studied in Krebs solution containing 1 mM tempol in vitro. The *mdx* + tempol in vivo group received 1 mM tempol in their drinking water for two weeks. Data were statistically compared by unpaired Student’s *t* tests with Welch’s correction where appropriate. $: Wild-Type vs. *mdx*; †: *mdx* vs. *mdx* Tempol in vitro; £: *mdx* vs. *mdx* Tempol in vivo.

**Table 2:** Overview of studies assessing antioxidant intervention in *mdx* mice.

Antioxidant	Author	Classification	Model	Age	Dose/Method of Delivery	Tissue Examined	Results
α-lipoic acid/L-carnitine	Hnia K. et al., 2007 [55]	Free radical scavenger	*mdx* mouse	5 weeks old	250 mg/kg α-lipoic acid/L-carnitine i.p injection for 14 days	Diaphragm	α-lipoic acid/L-carnitine decreased plasma CK levels and decreased muscle fibre central nucleation and fibre variance, antioxidant activity, lipid peroxidation, NF-kB and matrix metalloproteinase activity in *mdx* diaphragm. Β-dystroglycan expression was increased in *mdx* diaphragm following α-lipoic acid/L-carnitine.
Apocynin	Gonzalez D.R. et al., 2014 [24]	NADPH oxidase inhibitor	*mdx* cardiac myocytes	-	100 µM apocynin in vitro	Isolated cardiac myocytes	Apocynin restored contractility in *mdx* cardiac myocytes and normalised the amplitude of evoked intracellular Ca^2+^ concentration transients and total SR Ca^2+^ content. The production of spontaneous diastolic Ca^2+^ release events was decreased and SR Ca^2+^ leakage was decreased, thus apocynin improved SR Ca^2+^ handling and contractility in *mdx* cardiac myocytes.
Ascorbic acid (vitamin C)	Tonon E. et al., 2012 [54]	Antioxidant	*mdx* mouse	14 days old	Ascorbic acid 200 mg/kg via oral gavage daily for 14 days	Diaphragm	Ascorbic acid decreased plasma CK levels and diaphragm myonecrosis, inflammation, TNF-α and 4-HNE levels and Evans blue dye staining in *mdx* mice.
Cilostazol	Hermes Tde A.E. et al., 2016 [56]	PDE3 inhibitor	*mdx* mouse	14 days old	Cilostazol 100 mg/kg/day for 14 days	Diaphragm	Cilostazol reduced plasma CK and diaphragm myonecrosis, inflammatory cell area and macrophage infiltration, NF-kB and TNF-α content, ROS production and oxidative stress in *mdx* mice.
Diacerhein	Mâncio R.D. et al., 2017 [57]	IL-1β inhibitor	*mdx* mouse	14 days old	20 mg/kg/day diacerhein via oral gavage for 14 days	Diaphragm	Diacerhin reduced plasma CK levels, diaphragm muscle fibre damage and central nucleation, inflammatory mediators, oxidative stress and lipid peroxidation in *mdx* mice.
EUK-134	Kim J.H. and Lawler J.M. 2012 [53]	Superoxide dismutase mimetic	*mdx* mouse	20 days old	30 mg/kg/day EUK-134 i.p. injection for 8 days	Diaphragm	EUK-134 reduced 4-HNE, total hydroperoxides, positive staining of macrophages and T-cells, activation of NF-κB, p65 protein abundance and the number of centralised myonuclei and variability of fibre size in diaphragm muscle from *mdx* mice. Diaphragm contractile force was partially rescued following EUK-134 and increased citrate synthase activity in *mdx* mice.
Epigallocatechin-3-gallate (EGCG)	Nakae Y. et al., 2008 [58]	Green tea extract/antioxidant/Polyphenol	*mdx* mouse	From birth	5 mg/kg EGCG s.c. injection 4 times per week for 8 weeks	Diaphragm	EGCG had no effect on body weight and no observable toxic effects in the liver and kidney. EGCG decreased plasma CK and decreased the number of lipofuscin granules, necrotic muscle fibres and connective tissue in *mdx* diaphragm and increased utrophin expression. EGCG did not affect diaphragm isometric force.
SNT-NC17/Idebenone	Buyse G.M. et al., 2009 [59]	Antioxidant	*mdx* mouse	4 weeks old	200 mg/kg SNT-MC17/idebenone for 9 months	Heart	SNT-NC17/Idebenone corrected cardiac diastolic dysfunction, improved contractile reserve and voluntary running and decreased cardiac inflammation and fibrosis in *mdx* mice.
L-arginine	Marques M.J. et al., 2010 [60]	Amino acid	*mdx* mouse	6 months old	L-arginine in drinking water for 6 months	Heart	L-arginine had no effect on myocardial fibrosis but reduced the density of inflammatory cells in the *mdx* heart.
N-acetylcysteine (NAC)	Williams I.A. and Allen D.G. 2007 [19]	Glutathione precursor	*mdx* mouse	3 weeks old	1% NAC in drinking water for 6 weeks	Heart	NAC reduced DHE levels in *mdx* hearts, reduced abnormalities in *mdx* cardiomyocyte Ca^2+^ handling, returned *mdx* fractional shortening to WT values but did not affect Ca^2+^ sensitivity. NAC returned collagen type III and CD68 expression in *mdx* hearts to WT values.
N-acetylcysteine (NAC)	de Senzi Moraes Pinto R. et al., 2013 [61]	Glutathione precursor	*mdx* mouse	14 days old	150 mg/kg NAC i.p. daily for 14 days	Diaphragm	NAC reduced plasma CK levels and reduced TNF-α and 4-HNE protein adduct levels, inflammation, Evans blue dye staining and myonecrosis in *mdx* diaphragm muscle.
Resveratrol	Kuno A. et al., 2013 [62]	SIRT1 activator	*mdx* mouse	9 weeks old	4 g/kg resveratrol enriched diet for 32 weeks	Heart	Resveratrol downregulated the pro-hypertrophic co-activator p300 protein level in the *mdx* heart thus inhibiting fibre hypertrophy. Resveratrol also suppressed cardiac fibrosis and preserved cardiac diastolic function in *mdx* hearts.
Pentoxifylline	Gosselin L.E. and Williams J.E. 2006 [63]	PDE inhibitor	*mdx* mouse	4 weeks old	16 mg/kg/day pentoxyifylline for 4 weeks	Diaphragm	Pentoxyifylline had no effect on *mdx* diaphragm force, hydroxyproline concentration, type I and III procollagen mRNA and TGF-β mRNA.
Pentoxifylline	Burdi R. et al., 2009 [64]	PDE inhibitor	*mdx* mouse	4–5 weeks old	50 mg/kg/day pentoxyifylline i.p. injection for 4–8 weeks	Diaphragm	Pentoxifylline modestly increased *mdx* diaphragm isometric tetanic force.
Pyrrolidine dithiocarbamate (PDTC) or ursodeoxycholic acid(UDCA)	Graham K.M. et al., 2010 [65]	NF-κB inhibitors	*mdx* mouse	30 days old	50 mg/kg/day PDTC i.p. injection for one month 40 mg/kg/day UDCA i.p. injection for one month	Diaphragm	Neither PDTC or UDCA influenced collagen deposition or TGF-β1 expression in *mdx* diaphragm.
Quercetin	Hollinger K. et al., 2015 [66]	PGC-1α pathway activator	*mdx* mouse	3 months old	0.2% quercetin-enriched diet for 6 months	Diaphragm	Quercetin preserved diaphragm muscle fibres and reduced centralised nuclei, infiltrating immune cells, TNF-α gene expression and muscle fibrosis in *mdx* mice. Genes associated with oxidative metabolism were increased following quercetin.
Quercetin	Selsby J.T. et al., 2016 [67]	PGC-1α pathway activator	*mdx* mouse	2 months old	0.2% quercetin-enriched diet for 12 months	Diaphragm	Quercetin protected respiratory function in *mdx* mice during the first 4–6 months and declined thereafter. *Mdx* diaphragm muscle function and histology were not preserved following 12 months of quercetin treatment.
Quercetin	Ballmann C. et al., 2017 [68]	PGC-1α pathway activator	*mdx* mouse	2 months old	0.2% quercetin-enriched diet for 12 months	Heart	Quercetin decreased fibronectin, inflammation and indices of tissue damage while mitochondrial biogenesis and antioxidant enzymes were improved, and quercetin facilitated the assembly of the DAPC in *mdx* hearts.
Quercetin	Ballmann C. et al., 2015 [69]	PGC-1α pathway activator	*mdx* mouse	3 weeks old 3 months old	0.2% quercetin-enriched diet for 6 months	Heart	3 weeks old: Quercetin increased cytochrome-c and superoxide dismutase 2 protein expression, increased utrophin and decreased matrix metalloproteinase 9 abundance in *mdx* heart. 3 months old: Quercetin decreased relative and absolute heart weights, damage indicators and TGFβ-1 in *mdx* heart.
Sildenafil	Percival J.M. et al., 2012 [70]	PDE-5 inhibitor	*mdx* mouse	3 weeks old	400 mg/L sildenafil citrate in drinking water for 14 weeks	Diaphragm	Sildenafil modestly increased diaphragm force generating capacity and reduced fibronectin, TNF-α, matrix metalloproteinase 13 and Evans blue dye staining in the *mdx* diaphragm. Fatigue resistance and TGF-β were unaffected.
Vitamin E	Mancio R.D. et al., 2017 [71]	Peroxyl radical scavenger	*mdx* mouse	14 day old	40 mg vitamin E/kg daily via oral gavage for 14 days	Diaphragm	Vitamin E reduced muscle fibre damage, oxidative stress and inflammation processes in *mdx* diaphragm.

List of abbreviations: 4-HNE, 4-Hydroxynonenal; Ca^2+^, calcium; CD68, cluster of differentiation 68; CK, creatine kinase; DHE, dihydroethidium; EGCG, epigallocatechin-3-gallate; IL-1β, interleukin-1 beta; i.p., intra-peritoneal; MMP, matrix metalloproteinase; NAC, N-acetylcysteine; NADPH, nicotinamide adenine dinucleotide phosphate-oxidase; NFκB, nuclear factor lappa-light-chain-enhancer of activated B cells; PDE, phosphodiesterase; PDTC, pyrrolidine dithiocarbamate; PGC-1α, peroxisome proliferator activated receptor gamma co-activator 1 alpha; ROS, reactive oxygen species; SR, sarcoplasmic reticulum; s.c., sub-cutaneous; SIRT-1, sirtuin-1;TGF-β1, tumour growth factor beta 1; TNF-α, tumour necrosis factor alpha; UDCA, ursodeoxycholic acid.
